# Do Attachment Style and Emotion Regulation Strategies Indicate Distress in Predictive Testing?

**DOI:** 10.1007/s10897-015-9822-z

**Published:** 2015-10-01

**Authors:** Lucienne B. van der Meer, Erik van Duijn, Erik J. Giltay, Aad Tibben

**Affiliations:** 1 Department of Clinical Genetics, Leiden University Medical Center, PO Box 9600, 2300 RC Leiden, The Netherlands; 2 Department of Psychiatry, Leiden University Medical Center, PO Box 9600, 2300 RC Leiden, The Netherlands; 3 Center for Mental Health Care Delfland, Delft, The Netherlands; 4 Department of Clinical Genetics, Erasmus Medical Center, Rotterdam, The Netherlands

**Keywords:** Attachment style, Emotion regulation strategies, Predictive testing, Neurogenetic disorders, Huntington’s disease, CADASIL, HCHWA-D, Distress

## Abstract

Predictive genetic testing for a neurogenetic disorder evokes strong emotions, and may lead to distress. The aim of this study is to investigate whether attachment style and emotion regulation strategies are associated with distress in persons who present for predictive testing for a neurogenetic disorder, and whether these psychological traits predict distress after receiving test results. Self-report scales were used to assess attachment insecurity (anxiety and avoidance) and maladaptive emotion regulation strategies (self-blame, rumination, catastrophizing) in adults at 50 % risk for Huntington’s Disease (HD), Cerebral Autosomal Dominant Arteriopathy with Subcortical Infarcts and Leukoencephalopathy (CADASIL), and Hereditary Cerebral Hemorrhage With Amyloidosis - Dutch type (HCHWA-D), when they presented for predictive testing. Distress was measured before testing and twice (within 2 months and between 6 and 8 months) after receiving test results. Pearson correlations and linear regression were used to analyze whether attachment style and emotion regulation strategies indicated distress. In 98 persons at risk for HD, CADASIL, or HCHWA-D, attachment anxiety and catastrophizing were associated with distress before predictive testing. Attachment anxiety predicted distress up to 2 months after testing. Clinicians may consider looking for signs of attachment anxiety and catastrophizing in persons who present for predictive testing, to see who may be vulnerable for distress during and after testing.

## Introduction

Predictive genetic tests are available for a number of adult-onset autosomal dominant hereditary disorders, including neurogenetic disorders like Huntington’s Disease (HD), Cerebral Autosomal Dominant Arteriopathy with Subcortical Infarcts and Leukoencephalopathy (CADASIL), and Hereditary Cerebral Hemorrhage With Amyloidosis - Dutch type (HCHWA-D) (See Table 1).

**Table 1:** Clinical characteristics of Huntington’s disease, CADASIL, and HCHWA-D

	Huntington’s disease	CADASIL	HCHWA-D
Symptoms	progressive motor dysfunction, cognitive deterioration, psychiatric disturbances (Ross and Tabrizi 2011)	migraine with aura, multiple strokes, cognitive deterioration, psychiatric disturbances (Lesnik Oberstein et al. 2012)	recurrent hemorrhagic strokes, cognitive deterioration, dementia (Maat-Schieman et al. 2005)
Timing of clinical onset ^a^	Mid adulthood	Mid adulthood	Mid adulthood
Age of death	15–20 years from onset (Ross and Tabrizi 2011)	65 (Mean, yrs) (Herve and Chabriat 2010)	60 (Mean, yrs) (Maat-Schieman et al. 2005)
Treatment can alter onset or progression ^a^	No	No	No
Likelihood of development in gene mutation carriers ^a^	100 %	100 %	100 %

*CADASIL* Cerebral Autosomal Dominant Arteriopathy with Subcortical Infarcts and Leukoencephalopathy, *HCHWA-D* Hereditary Cerebral Haemorrhages with Amyloidosis - Dutch type

^a^Variables based on Family Systems Genetic Illness Model (Rolland and Williams 2005)

Asymptomatic individuals with a family history of one of these disorders can opt for predictive testing to find out whether or not they are carriers of the disease causing gene mutation. The impact of receiving a negative or positive test result is difficult to predict, and a case-by-case approach is recommended to meet the specific needs of the test applicant (Tibben 2007).

In most persons at risk for HD, CADASIL, or HCHWA-D, undergoing predictive testing evokes strong emotions, as the detection of a pathogenic gene mutation has major consequences for the individual as well as for family members. Since the introduction of predictive testing programs, clinicians and researchers have been interested in recognizing persons who may be vulnerable for distress during and after predictive testing. The risk of maladaptive psychological reactions to test results, in identified gene mutation carriers as well as in non-carriers, was found to be elevated in persons who have symptoms of depression or anxiety when entering the predictive testing program (Dufrasne et al. 2011; Pasacreta 2003; Tibben 2007), relatively high levels of neuroticism (Bennett et al. 2008; Reichelt et al. 2008), feelings of hopelessness about health (Bennett et al. 2008), limited ego strength (Tibben 2007; van Oostrom et al. 2007), or a passive, avoidant, or information-seeking coping style (Grosfeld et al. 2000; Tibben 2007; van Oostrom et al. 2007; Williams et al. 2010).

Attachment style and cognitive emotion regulation strategies are relevant for dealing with stressful situations and are associated with adjustment after emotional events (Mikulincer et al. 2006). In this study, adult attachment style and emotion regulation strategies are suggested as psychological traits allowing clinicians to gain more insight in the psychodynamics of persons who may be vulnerable for distress during and after predictive testing.

### Attachment Style

An attachment style is a set of mental representations of self and others in close relationships (Mikulincer et al. 2006; Pietromonaco et al. 2006), arising from different interaction patterns with parents or other attachment figures during childhood (Ainsworth et al. 1978; Bowlby 1973; Fraley and Shaver 2000; Mikulincer and Shaver 2003). An adult attachment style is *secure* when it is characterized by confidence in the availability of significant others in times of need and by comfort with closeness and interdependence (Pietromonaco et al. 2006). An adult attachment style is *insecure* when it is characterized by *attachment anxiety*, i.e., a tendency to worry about availability and responsiveness of significant others, fear of interpersonal rejection or abandonment, and an excessive need for approval from others (Brennan et al. 1998), and/or by *attachment avoidance*, i.e., a tendency to feel uncomfortable with interpersonal intimacy and dependency, an excessive need for self-reliance, and reluctance to self-disclose (Brennan et al. 1998). A personal attachment style is strongly linked to the way in which a person regulates unpleasant emotions (Mikulincer et al. 2006). Securely attached persons are generally resilient in stressful situations, as they foster optimistic representations of self and others, tend to mobilize social support, and manage to reduce distress by using constructive strategies of emotion regulation (Mikulincer et al. 2006; Sroufe 2005). Insecurely attached persons are more likely to interpret and evaluate events negatively, which leads to heightened stress levels (Pielage 2006) and increases the risk for emotional problems, maladjustment, and other psychological symptoms (Mikulincer et al. 2006).

Adult offspring of a parent with a neurogenetic disorder (HD, CADASIL, HCHWA-D) were previously found to show more adult attachment anxiety and poorer mental health, and to report more adversity in their childhood, especially in the form of parental dysfunction, compared to persons without one of these genetic diseases in their family (van der Meer et al. 2012, 2013). The level of attachment anxiety was associated with having experienced the parent’s disease process in childhood (van der Meer et al. 2013).

### Cognitive Emotion Regulation

Cognitive emotion regulation is a set of cognitive strategies that a person uses to manage uncomfortable emotions (Garnefski et al. 2001). Cognitive emotion regulation strategies are used during or after the experience of stressful events (Garnefski et al. 2001), such as predictive testing. Successful emotion regulation is essential for an individual’s personal and social life (Gross et al. 2006). A cognitive emotion regulation strategy that is considered to be adaptive in dealing with stressful events is *positive reappraisal*, i.e., attributing a positive meaning to a stressful event in terms of personal growth (Garnefski and Kraaij 2009). Using inadequate cognitive emotion regulation strategies, such as *self-blame* (i.e., blaming yourself for what you have experienced), *rumination* (i.e., excessive thinking about the feelings or thoughts associated with a negative event), and *catastrophizing* (i.e., explicitly emphasizing the terror of an experience) may result in maladjustment (Garnefski et al. 2001) or in psychopathology, such as depression and anxiety (Garnefski and Kraaij 2009).

Individuals with attachment anxiety tend to use emotion regulation strategies such as self-blame, rumination, and catastrophizing, and may thereby consolidate their feelings of distress (Pascuzzo et al. 2013). Individuals with attachment avoidance are likely to suppress their emotions and to seek little social support, thereby impairing their ability to deal with distress (Mikulincer et al. 2006; Pascuzzo et al. 2013).

The aim of this study is to investigate whether an insecure attachment style (attachment anxiety and attachment avoidance) and maladaptive emotion regulation strategies (self-blame, rumination, and catastrophizing) are associated with distress in persons who present for predictive testing for HD, CADASIL, or HCHWA-D, and whether these psychological traits predict distress after receiving (positive or negative) test results.

## Methods

### Participants

Adults (≥18 years) at 50 % risk for HD, CADASIL, or HCHWA-D were asked to participate in the study when they entered a predictive genetic testing program in the Leiden University Medical Center in Leiden (January 2008–January 2013), or in the Erasmus Medical Center in Rotterdam (January 2009–December 2009). A neurological exam was part of the predictive testing protocol; only persons found to be asymptomatic in this exam were asked to participate.

### Procedure

Predictive genetic testing was performed according to the predictive test guidelines for HD (MacLeod et al. 2013), in the Department of Clinical Genetics in either Leiden or Rotterdam. Persons at risk for HD, CADASIL, or HCHWA-D who requested predictive testing had a meeting with a clinical geneticist, a psychologist, and a neurologist, consecutively. After an interval of approximately 4 weeks they were seen by the same clinical geneticist and psychologist, and, if they decided to opt for testing, their blood was taken for DNA testing. Approximately 4 weeks later, they received the results of the predictive genetic test in a meeting with the clinical geneticist, and subsequently they had a meeting with the psychologist. Post-test psychological counseling was given according to the person’s needs.

After their first visit for predictive testing, eligible persons received oral and written information on the study, and, after informed consent, were given self-report scales. The study was approved by the Medical Ethics Committees of both participating hospitals.

Attachment style, cognitive emotion regulation strategies, and distress were assessed prior to receiving test results (baseline). To assess post-test distress, questionnaires were sent out in the week after receiving test results (T1) and approximately 26 weeks after receiving test results (T2). Reminders were given after 2 weeks (by mail) and after 4 weeks (by telephone). T1 was considered for analyses in this study when completed within 2 months (≤61 days) after receiving predictive test results; T2 was considered for analyses when completed between 6 and 8 months (≥183 days, ≤ 243 days) after receiving test results.

### Measurement

All instruments were self-report scales. Sociodemographic data (age, gender, marital status, education) were assessed using custom made survey items. Test result (negative or positive, i.e., pathogenic gene mutation absent or present) was collected from the medical files.

Distress before and after predictive testing was assessed using the Brief Symptom Inventory (BSI; 53 items, 5-point Likert-scale) (De Beurs and Zitman 2005; Derogatis 1975). The BSI measures nine primary symptom dimensions (somatization, obsessive-compulsive, interpersonal sensitivity, depression, anxiety, hostility, phobic anxiety, paranoid ideation, psychoticism). The mean of all items (BSI-Total, range 0–4) indicates current level of distress. Reliability and validity of the BSI are good (De Beurs and Zitman 2005). In the present study, the α-coefficient of the BSI-Total was 0.96. A validated reference value (95th percentile score) for the BSI-Total is 0.68 (Schulte-van Maaren et al. 2012).

Attachment style was assessed using the Experiences in Close Relationships-Revised (ECR-R; 36 items, 7-point Likert-scale) (Fraley et al. 2000). The ECR-R measures two dimensions of adult attachment, i.e., attachment anxiety (18 items, e.g., ‘I’m afraid that I will lose my partner’s love’, ‘I often wish that my partner’s feelings for me were as strong as my feelings for him or her’, ‘It makes me mad that I don’t get the affection and support I need from my partner’, range 1–7) and attachment avoidance (18 items, e.g., ‘I find it difficult to allow myself to depend on romantic partners’, ‘I don’t feel comfortable opening up to romantic partners’, ‘I am nervous when partners get too close to me’, range 1–7).

Higher attachment anxiety and/or attachment avoidance scores indicate a more insecure attachment style. Test-retest reliability of the ECR-R over 6 weeks is 86 % (Sibley and Liu 2004); α-coefficients in the present study were 0.93 for attachment anxiety, and 0.90 for attachment avoidance.

Cognitive emotion regulation strategies were assessed using the Cognitive Emotion Regulation Questionnaire (CERQ; 36 items, 5-point Likert-scale, ranging from ‘never’ to ‘always’) (Garnefski et al. 2001). The CERQ measures nine dimensions of cognitive emotion regulation, reflecting what people think after the experience of threatening or stressful life events. In this study, the CERQ was used as a general emotion regulation questionnaire, by asking how one copes with stressful situations in general. For this study, three subscales were used that are known to be associated with maladjustment in stressful circumstances: self-blame (4 items; e.g., “I think that basically the cause must lie within myself”), rumination (4 items; e.g., “I am preoccupied with what I think and feel about what I have experienced”), and catastrophizing (4 items; e.g., “I often think that what I have experienced is the worst that can happen to a person”). Subscale scores were obtained by summing the items of each subscale (range 0–16), indicating the extent to which a certain cognitive emotion regulation strategy is used. Reliability and validity of the CERQ are good (Garnefski et al. 2002). In the present study, α-coefficients were 0.78 for self-blame, 0.82 for rumination, and 0.82 for catastrophizing.

### Statistical Analysis

Data were analyzed with IBM SPSS Statistics 20 software (SPSS Inc., Chicago, IL, USA).

The course of distress over time (baseline, T1 and T2) was measured, using a *t*-test for independent samples to compare persons who had a positive predictive test result (carriers of the pathogenic gene mutation) to persons who had a negative result (non-carriers). Pearson correlations and linear regression were used to find associations between attachment style, cognitive emotion regulation, and distress during and after predictive testing. The BSI-Total at baseline (log transformed to attain a normal distribution of data) was used as the dependent variable. The independent variables entered into the regression were gender, age, and the psychological traits attachment anxiety, attachment avoidance, self-blame, rumination, and catastrophizing. This procedure was repeated for T1 and T2 using the log transformed BSI-Total as the dependent variable. In the T1 and T2 analyses, predictive test result (negative or positive) and distress at baseline (BSI-Total) were added to the set of independent variables. Finally, as the three time points were not independent, multilevel regression analysis (i.e., linear mixed models) was used (with a compound symmetry covariance matrix) to investigate the independent contribution of baseline variables on distress at follow-up, adjusted for all other potential predictors in one multivariable model. The two-level structure consisted of the three time points (i.e., lower level) and the subjects (i.e., higher level). All tests were two-tailed, with *p* < 0.05 denoting statistical significance.

## Results

### Descriptives of Participants

**Fig. 1:**
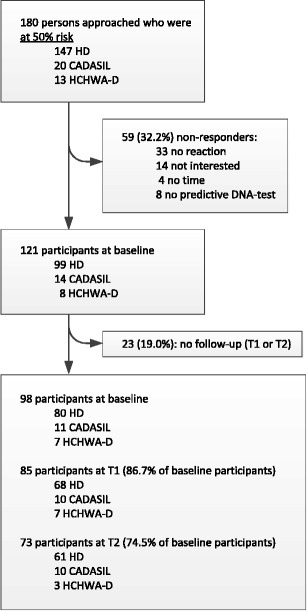
Flowchart of participants. Baseline = baseline assessment, prior to receiving DNA test results. T1 = first follow-up assessment; median 16 days after receiving DNA test results. T2 = second follow-up assessment; median 193 days after receiving DNA test results

A total of 180 persons were approached for the study. Of those, 51 (28.3 %) did not complete the baseline assessment, and 8 (4.4 %) declined predictive DNA-testing during the program. A total of 59 persons were therefore not included in analyses (See Fig. 1).

Of the 121 persons who underwent predictive testing and completed the baseline assessment, 23 (19 %) did not complete either of the post-test assessments (T1 or T2) and were therefore not included in this study. These individuals were significantly younger than participants, but did not differ from participants in sex, education level, marital status, or test result status. In total, 45.6 % of the persons who were approached could not be included.

Of the 98 included participants, 80 (81.6 %) were at risk for HD, 11 (11.2 %) for CADASIL, and 7 (7.1 %) for HCHWA-D. Mean age of participants was 37.3 years (range 19–69, SD 12.3), 48 (49.0 %) were male, 83 (84.7 %) were in a relationship with a partner, and 47 (48.0 %) had 11 or more years of education.

T1 was completed in time by 85 participants (86.7 %), at a median duration of 16 days (range 2–53 days) after receiving test results; 5 participants (5.1 %) completed T1 too late and 8 participants (8.1 %) did not respond at T1.

T2 was completed in time by 73 participants (74.5 %), at a median duration of 194 days (range 183–243 days) after receiving test results; 13 participants (13.3 %) completed T2 too late and 12 participants (12.2 %) did not respond at T2.

### Distress Level at Baseline and After Predictive Testing

**Fig. 2:**
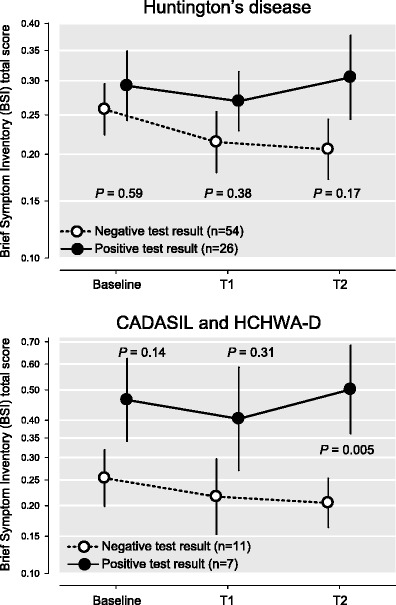
Distress scores of participants with negative or positive test result (Huntington’s Disease, CADASIL and HCHWA-D). CADASIL = Cerebral Autosomal Dominant Arteriopathy with Subcortical Infarcts and Leukoencephalopathy. HCHWA-D = Hereditary Cerebral Haemorrhages with Amyloidosis - Dutch type. The BSI scores are depicted on a logarithmic scale because of its positively skewed distribution. Differences between the two groups per time point are tested using a *t*-test for independent samples

Median distress scores of all participants were low at baseline (0.30, Interquartile range (IQR) 0.13–0.47) and remained low at T1 (0.23, IQR 0.11–0.48), and at T2 (0.25, IQR 0.09–0.49). Persons who received a positive test result (carriers; *n* = 33) had a median distress score of 0.32 at baseline (IQR 0.19–0.56), 0.24 at T1 (IQR 0.15–0.50), and 0.35 at T2 (IQR 0.17–0.68), whereas persons who received a negative test result (non-carriers, *n* = 65) had a median distress score of 0.28 at baseline (IQR 0.12–0.46), 0.21 at T1 (IQR 0.06–0.45), and 0.19 at T2 (IQR 0.08–0.40). There were no significant differences in distress between carriers and non-carriers at baseline or T1. However, at T2 carriers were more distressed at than non-carriers (*t* = 2.45, *p* = 0.02) (See Fig. 2).

### Associations Between Attachment Style, Cognitive Emotion Regulation, and Distress Before and After Testing

#### Baseline (Before Testing)

Univariate regression analysis showed that the level of distress at baseline was strongly associated with all psychological traits (attachment anxiety, attachment avoidance, self-blame, rumination, and catastrophizing; beta-values between 0.282 and 0.472, *p*-values between 0.005 and < 0.001; see Table 2). In multivariate analysis, the level of distress at baseline was strongly associated with attachment anxiety (beta = 0.239, *p* = 0.03), and catastrophizing (beta = 0.241, *p* < 0.02), and there was a borderline significance with self-blame (beta = 0.181, *p* = 0.056),

**Table 2:** Variables associated with distress before and after predictive testing in persons at risk; linear regression

	Distress ^a^ at baseline (*n* = 98)				Distress ^a^ at T1 (*n* = 85)				Distress ^a^ at T2 (*n* = 73)				Multilevel (MIXED) analysis (*n* = 98)	
Associations	Univariate		Multivariate		Univariate		Multivariate		Univariate		Multivariate		Multivariate	
	beta	*p*	beta^a^	*p*	beta	*p*	beta^a^	*p*	beta	*p*	beta^a^	*p*	beta^a^	*p*
Female sex (ref. male)	0.093	0.36	0.088	0.29	0.055	0.62	0.009	0.90	0.168	0.15	0.001	0.99	0.044	0.70
Age (yr)	−0.040	0.70	−0.046	0.61	−0.073	0.51	0.078	0.29	0.033	0.78	0.050	0.56	0.040	0.51
Positive test result (ref. negative result)					0.140	0.20	0.062	0.37	0.274	0.02	0.236	0.007	0.226	0.07
BSI-Total at baseline (score)					0.789	<0.001	0.771	<0.001	0.723	<0.001	0.717	<0.001	0.787	<0.001
Attachment / cognitive emotion regulation styles:														
Attachment anxiety	0.472	<0.001	0.239	0.03	0.501	<0.001	0.218	0.02	0.335	0.004	−0.165	0.17	0.052	0.50
Attachment avoidance	0.282	0.005	0.131	0.21	0.219	0.045	−0.063	0.45	0.329	0.005	0.173	0.13	0.043	0.56
Self-blame	0.382	<0.001	0.181	0.056	0.342	0.001	−0.015	0.85	0.215	0.07	0.072	0.45	−0.007	0.92
Rumination	0.411	<0.001	0.138	0.20	0.364	0.001	0.024	0.78	0.211	0.07	−0.029	0.80	0.036	0.66
Catastrophizing	0.461	<0.001	0.241	0.02	0.269	0.01	−0.161	0.06	0.315	0.007	0.000	1.000	−0.088	0.22

^a^ Distress defined as the mean of all items of the Brief Symptom Inventory (log transformed)

BSI-Total = Mean of all items of the Brief Symptom Inventory (log transformed)

#### After Testing

In a univariate regression analysis, the level of distress at T1 was associated with baseline distress (beta = 0.789, *p* < 0.001) and with all psychological traits (beta-values between 0.219 and 0.501, *p*-values ranging from 0.045 to < 0.001; see Table 2).

In a multivariate regression analysis, the level of distress at T1 was predicted by baseline distress (beta = 0.771, *p* < 0.001) and by attachment anxiety (beta = 0.218, *p* = 0.02). When testing for the change in *R* ^2^, indicative of the explained variance, we found that baseline distress accounted for 61.3 % of variance of the level of distress at T1, attachment anxiety accounted for an additional 2.3 % of explained variance.

At T2, univariate regression analysis showed that the level of distress was associated with baseline distress (beta = 0.723, *p* < 0.001), and by the psychological traits attachment anxiety, attachment avoidance, and catastrophizing (beta-values between 0.315 and 0.335, *p*-values ranging from 0.004 to 0.007). Multivariate analysis showed that the level of distress at T2 was predicted by baseline distress (beta = 0.717, *p* < 0.001) and by having a positive test result (beta = 0.236, *p* = 0.007). Again, baseline distress accounted for the majority of 52.2 % of variance of the level of distress at T2, while a positive test result accounted for an additional 4.6 % of explained variance. The multilevel analysis, that took both T1 and T2 assessments into account, showed that post-test distress was predicted by baseline distress (beta = 0.787, *p* < 0.001) and a positive test result (beta = 0.226, *p* = 0.07).

## Discussion

Do attachment style and emotion regulation strategies indicate distress in predictive testing?

In a group of 98 persons who underwent predictive testing for an adult onset autosomal dominant neurogenetic disorder (HD, CADASIL, or HCHWA-D), it was found that persons with relatively high levels of attachment anxiety or catastrophizing were more likely to report higher distress levels when they entered the predictive testing process. Individuals with attachment anxiety were also more distressed up to 2 months after receiving test results. Attachment avoidance and maladaptive emotion regulation strategies did not add significantly to the prediction of distress after disclosure of test results.

This study is the first to relate attachment style and strategies for emotion regulation to the level of distress that individuals experience during a predictive testing process. Given the relevance of attachment style and emotion regulation strategies for dealing with stressful situations, the results of this study may enhance understanding of individual reactions to predictive testing.

### Distress Levels at Baseline and After Predictive Testing

Most participants, carriers as well as non-carriers, had relatively low levels of distress, before and after the predictive test. In general, and in accordance with what is known from previous studies (Dufrasne et al. 2011; Pasacreta 2003; Tibben 2007) it can be stated that persons who are distressed before the predictive test will be equally distressed after receiving test results, and that those who are not distressed before the test, will continue to have a low level of distress after receiving test results. Test status (i.e., positive vs negative) alone does not explain why someone experiences distress after testing. A person’s psychological make-up, in terms of attachment style or emotion regulation style, may influence the level of distress both before and after testing.

### Associations Between Attachment Style, Cognitive Emotion Regulation, and Distress Before Testing

The finding that distress at baseline (before testing) was associated with attachment anxiety, catastrophizing, and, although only marginally, self-blame, is in accordance with previous research and with the supposition that attachment style and cognitive emotion regulation are relevant for dealing with stressful situations and for regulating unpleasant emotions (Mikulincer et al. 2006).

Individuals with relatively high levels of attachment anxiety are known to report prolonged and uncontrollable negative thoughts (rumination), and tend to cope with distress by exaggerating it (catastrophizing), which can result in intense emotions and in some cases depression (Mikulincer et al. 2006; Pietromonaco et al. 2006; Wei et al. 2003). Individuals with relatively high levels of attachment anxiety may find it difficult to rely on their partner, relatives, and friends, fearing that others will be unable to respond to their needs, or even reject or abandon them after a positive test result (Mikulincer and Shaver 2008). Possibly, individuals with relatively high levels of attachment anxiety have more difficulty deciding whether to undergo predictive testing, fearing they will not be able to cope with the distress of the test, or fearing a lack of social support during testing or after receiving a positive test result. Future studies are needed to investigate the relationship between attachment style and decision making about predictive testing.

According to the present study, attachment avoidance is not related to distress during the predictive testing process. It should be noted, however, that individuals with relatively high levels of attachment avoidance may find it difficult to express their thoughts and emotions, or may not be aware of having problems, and are known to underreport symptoms of distress (Mikulincer and Shaver 2008). Individuals with relatively high levels of attachment avoidance may not seek support from friends, relatives, or even their partner, which may impair their ability to deal with the emotions of predictive testing and may result in emotional problems (Mikulincer et al. 2006; Pietromonaco et al. 2006).

Earlier research showed that in adult offspring of a parent with HD, CADASIL, or HCHWA-D, attachment insecurity, in particular attachment anxiety, was more prevalent than in a non-clinical reference group, which may be related to a childhood that was negatively influenced by the parent’s disease (van der Meer et al. 2013). Individuals presenting for predictive testing for one of these disorders may therefore have an elevated chance of having attachment anxiety, and, as the present study indicates, may be relatively likely to experience distress during the testing process.

The finding that persons who accentuate and exaggerate their unpleasant emotions experience relatively high levels of distress when entering the predictive testing process is in accordance with previous studies on emotion regulation in other domains. The use of maladaptive emotion regulation strategies, such as self-blame and catastrophizing, may increase vulnerability to developing psychopathology (e.g., depression and anxiety) in response to stressful events (Garnefski and Kraaij 2009), such as undergoing predictive testing. In a context of predictive testing, catastrophizing may take the form of interpreting normal phenomena as symptoms of the neurogenetic disease, or having recurrent thoughts of terrible things that may happen in the future due to the neurogenetic disease. Self-blame, which was marginally associated with distress, may imply feeling guilty towards offspring, family members, or a partner, for being at risk for a neurogenetic disease, or for possibly having transmitted a genetic disorder (or mutation) to children.

### Associations Between Attachment Style, Cognitive Emotion Regulation, and Distress After Testing

The strongest predictor of post-test distress was the level of distress before testing, which was also found in previous studies (Dufrasne et al. 2011; Pasacreta 2003; Tibben 2007). Individuals with higher levels of attachment anxiety experienced more distress in the first 2 months after receiving test results. Persons with relatively high levels of attachment anxiety may lack confidence in their own capacity to cope with the test result and may have difficulty calling on others for support when they experience distress after testing, out of fear of being rejected or disappointed. This may prevent adequate distress reduction after testing. More than 6 months after testing, attachment anxiety is no longer associated with distress. Apparently, persons with high attachment anxiety are more distressed after receiving predictive test results than others, but they do adapt over time. Future research should investigate which factors contribute to distress reduction in persons with high attachment anxiety in the months after receiving predictive test results.

In contrast to what might be expected based on previous studies (Garnefski et al. 2001; Garnefski and Kraaij 2009; Mikulincer et al. 2006; Pielage 2006), attachment avoidance and the maladaptive emotion regulation strategies rumination, self-blame, and catastrophizing did not contribute to the strong association of pretest distress with distress after predictive testing. This may in part be explained by the strong correlation between these variables and the level of distress before testing.

### Practice Implications

Psychological counseling in predictive testing programs involves exploring the at-risk person’s coping strategies and current emotions, such as anxiety, depressive feelings, and uncertainty. Knowledge on attachment theory and related phenomena like emotion regulation strategies may help clinicians understand the psychodynamics of highly distressed individuals and may guide them in selecting tools that promote reduction of distress during and after testing.

This study’s findings indicate that clinicians involved in predictive testing (e.g., clinical geneticists, genetic counselors, psychologists, or social workers) may consider identifying test applicants with attachment anxiety and maladaptive emotion regulation strategies, as these individuals are vulnerable for distress. Psychological traits like attachment style and emotion regulation strategies do not show much fluctuation over time (in contrast to psychological states like distress), which allows identification of these characteristics at any given time.

Early identification of these characteristics in test applicants, especially in those who seem to be particularly distressed, may help guide appropriate interventions to promote adequate adjustment and to prevent or alleviate distress during and after the predictive test. This would do justice to the call for a case-by-case approach in predictive testing (Tibben 2007). Persons with high levels of attachment anxiety may benefit from one or more pre-test sessions with a sensitive and responsive counselor, allowing them to feel safe and supported enough to be able to deal with test results.

Test applicants’ social support and their expectations of others during and after testing should also be explored by clinicians, preferably before testing. The support of a partner or other security-providing person is likely to help most test applicants feel secure throughout the stressful test period, and may prevent distress (Mikulincer et al. 2006; Pietromonaco et al. 2006). The predictive test guidelines for HD (MacLeod et al. 2013) state: “The participant should be encouraged to select a companion to accompany him/her throughout all the different stages: the pre-test, the taking of the test, the delivery of the results and the post-test stage” (Recommendation 3). However, persons with high levels of attachment anxiety may find it difficult to rely on their partner or a friend, fearing a lack of support or an untoward reaction, whereas persons with high levels of attachment avoidance may not feel they need anyone to support them during the test process. Persons who present for testing without a companion, possibly due to attachment anxiety of attachment avoidance, may benefit from additional psychological counseling before or after testing, to allow them to feel secure and to share their thoughts and worries, in order to prevent or reduce distress.

More adequate coping in persons who have trouble regulating their emotions during the predictive testing process may be promoted by addressing and challenging negative thoughts, and by stimulating adaptive emotion regulation strategies, such as ‘positive reappraisal’, which refers to attributing a positive meaning to an event in terms of personal growth (Garnefski et al. 2002; Garnefski and Kraaij 2009). Clinical experience shows that, even in the difficult situation of facing a future with a neurogenetic disease, it is possible to formulate new life goals (e.g., searching a more satisfying job, traveling, etc.), or underline positive aspects (e.g., ‘This makes us stronger as a couple’, ‘The positive test result allows me to make arrangements for what lies ahead’). Persons with more negative thoughts may be supported in looking for positive aspects that help them cope better. Positive reappraisal is known to be positively related to measures of optimism and self-esteem and to the presence of less symptoms of psychopathology (Garnefski and Kraaij 2006; Garnefski et al. 2002).

### Research Recommendations

Future studies are needed to determine to what extent secure attachment styles and adaptive emotion regulation strategies are associated with distress after predictive testing. Furthermore, it would be interesting to investigate whether persons at risk for HD, CADASIL, or HCHWA-D who do not present for predictive testing have a different attachment style and/or tend to use different emotion regulation strategies compared to those who opt for testing. Individuals who are vulnerable for distress due to attachment insecurity or a maladaptive style of emotion regulation may be reluctant to present for predictive testing.

This study focusses on genetic disorders that will certainly develop in gene mutation carriers, and for which there is currently no cure (See Table 1). Future studies are needed to determine whether attachment style and emotion regulation strategies play a comparable role in predictive testing for disorders with a lower likelihood of development in gene mutation carriers and for which there are preventive or treatment options, such as hereditary cancer syndromes.

### Study Limitations

Several limitations of this study should be mentioned. First, the time range in which participants completed T1 and T2 was somewhat broad. In spite of the request to send back the questionnaires within 2 weeks after receiving them, some participants responded much later. The results of T1 and T2 should therefore not be interpreted as reflecting specific time points, but rather as indications of distress in the weeks and months after testing. Second, few eligible persons at risk for CADASIL or HCHWA-D were included, because these disorders are very rare and few people request predictive testing. However, because of the paucity of information on predictive testing for these disorders, it was considered important to include these groups. Third, although participants underwent a neurological exam and were found to be without symptoms, some participants may have had subtle psychiatric or cognitive symptoms (Van Duijn et al. 2008), which may have influenced self-rating. Fourth, all instruments were self-report scales, which may have caused measurement error and social desirability bias. Finally, external validity is reduced by the fact that a substantial part of the persons who were approached for the study could not be included.

## Conclusions

Persons at risk for a neurogenetic disease who have relatively high levels of attachment anxiety and who tend to catastrophize are more likely to be distressed when they present for predictive testing. Individuals with an anxious attachment style will continue to be distressed in the first 2 months after testing, largely independent of their test result. These findings may support clinicians working in predictive testing programs in identifying persons who may be vulnerable for distress and in choosing appropriate interventions to promote stress reduction.
